# Derivation of Dirac equation from the stochastic optimal control principles of quantum mechanics

**DOI:** 10.1038/s41598-024-56582-5

**Published:** 2024-03-18

**Authors:** Vasil Yordanov

**Affiliations:** Faculty of Physics, Sofia University, 5 James Bourchier Blvd., 1164 Sofia, Bulgaria

**Keywords:** Quantum mechanics, Theoretical physics

## Abstract

In this paper, we present a stochastic approach to relativistic quantum mechanics. We formulate the three fundamental principles of this theory and derive the Dirac equations based on them. This approach enables us to bring more insight into the nature of Dirac’s spinors. Furthermore, we provide a physical interpretation of the stochastic optimal control theory of quantum mechanics.

## Introduction

The connection between stochastic optimal control theory and quantum mechanics is profound, with a long history^1,2^. Its roots can be traced back to the pioneering works of Fürth^3,4^, Fényes^5^ and stochastic mechanics of Nelson^6^, and it is even linked to Feynman’s path integral formulation of quantum mechanics^7,8^. Later in the 1980s, Yasue^9^ formulated the variational principle in stochastic mechanics, while Guerra and Morato^10^ applied stochastic control theory. Despite its rich history, the theory continues to evolve and expand^11–14^.

Stochastic optimal control theory of quantum mechanics attempts to give a realistic, objective description of physical events in the microphysical world aiming to bridge the gap between classical and quantum interpretations. Notably, it represents physical observables through stochastic processes instead of self-adjoint operators^15^.

Unlike traditional quantum mechanics, which postulates the Schrödinger equation, this theory derives it from three underlying principles:

1. Particles move as Brownian particles in four-dimensional spacetime, influenced by an external random spacetime force^6^.
2. The stochastic movement of the particle turns its classical action integral into a stochastic variable.
3. Nature tries to minimize the expected value for the action, in which the particle’s velocity is consider to be a control parameter of the optimization.

The principles formulated above are not new in this paper. However, attributing them to a single author would not be correct, as they have developed over time with contributions from various scientists^9,10,12,16,17^.

Building on these fundamental principles, the theory allows us not only to derive the Schrödinger equation but also to explain the Born rule and the operator substitution rule^12^.

There are a variety of approaches ^18^ to the derivation of the Dirac equation, but none of them is based on stochastic optimal control theory. Although there have been other investigations that aim to construct a stochastic optimization theory for relativistic quantum mechanics^12,19–22^, none of them have succeeded in deriving the Dirac equation or in explaining the nature of the Dirac spinor. In this paper, we extend the stochastic optimal control theory of quantum mechanics to also derive the Dirac equation^23^ and to provide new insights into the nature of Dirac’s four-component spinor $\psi$.

1 $$\begin{aligned} \gamma ^\mu (i \hbar \partial _\mu - e A_\mu ) \psi = m c \psi , \end{aligned}$$

where $\gamma ^\mu$ are 4x4 Dirac matrixes with anticommutation rules: $\{ \gamma ^\mu , \gamma ^\nu \} = 2 g^{\mu \nu } {\mathbb {I}}_4$, here the ${\mathbb {I}}_4$ is the identity four-by-four matrix, and $g^{\mu \nu }$ is the metric tensor of spacetime.

We will take advantage of the standard notation for contravariant and covariant tensors, as well as the Einstein summation convention.

## Stochastic optimal control theory of quantum mechanics

Let us express the previously mentioned fundamental principles of Stochastic Optimal Control Theory of quantum mechanics in mathematical terms.

The stochastic equation of motion of the Brownian particle^24^ is:

2 $$\begin{aligned}dx_\mu =u_\mu ds+ \sigma _\mu dW_\mu , \quad \mu =0..3, \end{aligned}$$

where $x_\mu$ denote the spacetime coordinates ${{\textbf {x}}}$ of the particle, and $u_\mu$ represents the components of the four-velocity ${\textbf{u}}$. In the context of applying optimal control theory, the velocity $u_\mu$ is referred to as the Markov control policy. The $W_\mu$ terms are standard independent Wiener processes, and $\sigma _\mu$ represent the diffusion coefficients.

Note that in Eq. (2), we consider diffusions not only in three spatial dimensions but also allow the time variable to undergo a diffusion process. This assumption puts the time dimension on equal footing with the spatial dimensions and is in line with the principles of special relativity, which combines space and time into a single entity of spacetime. The concept of temporal diffusion was introduced in^12,20^.

The action is postulated to be the minimum of the expected value of the stochastic action:

3 $$\begin{aligned} S({\textbf{x}}_i, {\textbf{u}}(\tau _i \rightarrow \tau _f))= \min _{\textbf{u}(\tau _i \rightarrow \tau _f)} \left\langle \int _{\tau _i}^{\tau _f} ds\, {\mathcal {L}}({\textbf{x}}(s), {\textbf{u}}(s),s) \right\rangle _{{\textbf{x}}_i}, \end{aligned}$$

where ${\mathcal {L}}({\textbf{x}}(s), {\textbf{u}}(s), s)$ is the Lagrangian of the test particle, which is a function of the control policy ${\textbf{u}}(s)$ and the four-coordinates ${\textbf{x}}(s)$ at proper time *s*. Note that *s* need not be the proper time; it can be any monotonic function representing the progress of the world point along the particle’s world line (see^25^, Chapter 7.10). The subscript ${\textbf{x}}_i$ on the expectation value indicates that the expectation is taken over all stochastic trajectories that start at ${\textbf{x}}_i$.

The task of optimal control theory, as described by Bellman^26^, is to find the control ${\textbf{u}}(s)$ for the time interval $\tau _i< s < \tau _f$, often denoted as ${\textbf{u}}(\tau _i \rightarrow \tau _f)$, that minimizes the expected value of the action $S({\textbf{x}}_i, {\textbf{u}}(\tau _i \rightarrow \tau _f))$.

We introduce the optimal action for any intermediate proper time $\tau$ such that $\tau _i< \tau < \tau _f$:

4 $$\begin{aligned} J(\tau , {\textbf{x}}_\tau )=\min _{{\textbf{u}}(\tau \rightarrow \tau _f )} \left\langle \int _{\tau }^{\tau _f} ds\, {\mathcal {L}}(s, {\textbf{x}}_s, {\textbf{u}}_s) \right\rangle _{ {\textbf{x}}_\tau }, \end{aligned}$$

in stochastic optimal control theory, this function is commonly referred to as the “cost-to-go” function, and the use of the symbol “*J*” as notation for it is a common convention.

By definition, the action $S({\textbf{x}}_i, {\textbf{u}}(\tau _i \rightarrow \tau _f))$ is equal to the cost-to-go function $J(\tau _i, {\textbf{x}}_{\tau _i})$ at the initial proper time and spacetime coordinate:

5 $$\begin{aligned} S({\textbf{x}}_i, {\textbf{u}}(\tau _i \rightarrow \tau _f))=J(\tau _i, {\textbf{x}}_{\tau _i}). \end{aligned}$$

In following papers^27–30^ one can find the derivation of the Hamilton–Jacobi–Bellman (HJB) equation, which is a partial differential equation for $J(\tau , {\textbf{x}})$ with boundary conditions $J(\tau _f, {\textbf{x}}) = 0$. The final result for this equation is:

6 $$\begin{aligned} -\partial _\tau J(\tau , {\textbf{x}})=\min _u \left( {\mathcal {L}}(\tau , {\textbf{x}}, {\textbf{u}}) + u^\mu \partial _\mu J(\tau , {\textbf{x}}) + \frac{1}{2} \sigma ^{\mu } \sigma ^\mu \partial _{\mu \mu } J(\tau , {\textbf{x}}_\tau ) \right) . \end{aligned}$$

The optimal control at the current ${\textbf{x}}$, $\tau$ is given by:

7 $$\begin{aligned} u({\textbf{x}}, \tau ) = \arg \min _{\textbf{u}} \left( {\mathcal {L}}(\tau , {\textbf{x}}, {\textbf{u}}) + u^\mu \partial _\mu J(\tau , {\textbf{x}}) \right) . \end{aligned}$$

To describe a quantum system, one must start with a given Lagrangian for the system and then solve the Eq. (6).

## Covariant relativistic Lagrangians

In Eq. (6), we employ the following Lorentz-invariant Lagrangian for a single particle in an electromagnetic field, as detailed in^25^.

8 $$\begin{aligned} \Lambda = {\tilde{\sigma }} m c \sqrt{{\tilde{\sigma }} u_\mu u^\mu } + q A_\mu u^\mu , \end{aligned}$$

where *q* represents the charge of the particle and $A_\mu$ denotes the 4-vector potential. The symbol ${\tilde{\sigma }}$ indicates the sign convention for the metric tensor: it takes the value of $+1$ for the metric with diagonal elements $(1,-1,-1,-1)$ and $-1$ for the metric $(-1,1,1,1)$, as elaborated in^31^.

The components of the four-velocity of the particle are related to the speed of light by the equation:

9 $$\begin{aligned} u_\mu u^\mu = {\tilde{\sigma }} c^2. \end{aligned}$$

This relation, referred to as the “weak equation” by Dirac, allows us to treat $u^\mu$ as unconstrained quantities until all differentiation operations have been carried out, at which point we impose the condition of Eq. (9) (see^25^ Chapter 7.10). This will be the approach we employ as we seek to minimize the expected value of the stochastic action.

## Derivation of Dirac equation

Let us find the minimum of the expected value of the stochastic action using the Lagrangian (8). Let us substitute this Lagrangian in (6):

10 $$\begin{aligned} -\partial _\tau J(\tau , {\textbf{x}})=\min _u \left( {\tilde{\sigma }} mc \sqrt{{\tilde{\sigma }} u_\mu u^\mu } + u^\mu \left( \partial _\mu J(\tau , {\textbf{x}}) + q A_\mu \right) + \frac{1}{2} \sigma ^\mu \sigma ^\mu \partial _{\mu \mu } J(\tau , {\textbf{x}}) \right) . \end{aligned}$$

If we take derivative with respect to $u^\mu$ we can find the optimal control policy $u^\mu$ at which the above expression has minimum:

11 $$\begin{aligned} {\tilde{\sigma }} c \frac{2 {\tilde{\sigma }} u^{\min}_\mu }{2 \sqrt{{\tilde{\sigma }} u^{\min}_\mu u^\mu _{\min}}} + \frac{1}{m} \left( \partial _\mu J(\tau , {\textbf{x}}) + q A_\mu (\tau , {\textbf{x}}) \right) = 0. \end{aligned}$$

If we use the weak condition (9) then we derive:

12 $$\begin{aligned} u^{\min}_\mu + \frac{1}{m} \left( \partial _\mu J(\tau , \textbf{x}) + q A_\mu (\tau , {\textbf{x}}) \right) = 0. \end{aligned}$$

For short in the rest of this paper we will denote with $u_\mu$ the optimal control policy and will skip the superscript of $u^{\min}_\mu$.

13 $$\begin{aligned} u_\mu = - \frac{1}{m} \left( \partial _\mu J(\tau , {\textbf{x}}) + q A_\mu (\tau , {\textbf{x}}) \right) \end{aligned}$$

We want to linearise Eq. (10) with respect to $u_\mu$. For this purpose we will use similar approach that Dirac^23^ has used to linearise the relativistic energy. The idea of the linearization of the Lagrangian in HJB equation is the main idea in this paper and we will see that this idea will lead us to the derivation of Dirac equation. We write:

14 $$\begin{aligned} {\tilde{\sigma }} m c \sqrt{{\tilde{\sigma }} u_\mu u^\mu } = m c \gamma ^\mu u_\mu , \end{aligned}$$

here, the $\gamma ^\mu$ are the Dirac matrices^23^. Note that since $\gamma ^\mu$ is a four-by-four matrix, we should assume that the left-hand side of this equation is multiplied by a four-by-four matrix whose square is the identity matrix.

Since the four-velocity is optimal, we can then remove the minimization function from (10) and use (14) to obtain:

15 $$\begin{aligned} -\partial _\tau J(\tau , {\textbf{x}})=mc \gamma ^\mu u_\mu + u^\mu \left( \partial _\mu J(\tau , {\textbf{x}}) + q A_\mu (\tau , {\textbf{x}}) \right) + \frac{1}{2} \sigma ^\mu \sigma ^\mu g_{\mu \mu } \partial ^\mu \partial _\mu J(\tau , {\textbf{x}}), \end{aligned}$$

here, we have raised the index of the derivative in the last term and assumed summation over $\mu$ in this term.

In equation (15), $\gamma ^\mu$ is a four-by-four matrix. To align the dimensions of each term, we assume multiplication by a four-by-four matrix whose square is the identity matrix. To find the appropriate matrix, we will consider the example of a particle at rest with four-velocity (*c*, 0, 0, 0). In this case, the equation simplifies to: $-\partial _\tau J(\tau , \textbf{x})=mc^2 \gamma ^0 - m c^2$. It is clear now that we need to multiply the scalar terms by $\gamma ^0$. If we use the identities $\gamma ^0 \gamma ^0={\mathbb {I}}_4$, ${\mathbb {I}}_4 {\mathbb {I}}_4={\mathbb {I}}_4$, and multiply both sides of  (15) by $\gamma ^0$, then we obtain:

16 $$\begin{aligned}- \partial _\tau J(\tau , {\textbf{x}}) {\mathbb {I}}_4= mc \gamma ^\mu u_\mu \gamma ^0 + u^\mu {\mathbb {I}}_4 \left( \partial _\mu J(\tau , {\textbf{x}}) + q A_\mu (\tau , {\textbf{x}}) \right) {\mathbb {I}}_4 + \frac{1}{2} \sigma ^\mu \sigma ^\mu g_{\mu \mu } \partial ^\mu \partial _\mu J(\tau , {\textbf{x}}) {\mathbb {I}}_4. \end{aligned}$$

Now we will introduce the following notation:

17 $$\begin{aligned} \epsilon _r = {\left\{ \begin{array}{ll} 1 &{} \quad \text {if } r=1,2 \\ -1 &{}\quad \text {if } r=3,4 \end{array}\right. }. \end{aligned}$$

Let us also denote $\gamma ^0_{(r)}$ and ${\mathbb {I}}^{(r)}_4$, where $r=1,2,3,4$, as the $r^{\text{th}}$ column of the $\gamma ^0$ matrix and the identity matrix ${\mathbb {I}}_4$, respectively. It is obvious that $\gamma ^0_{(r)} = \epsilon _r {\mathbb {I}}^{(r)}_4$ for $r=1,2,3,4$. We will consider Eq. (16) as four independent equations, one corresponding to each column of the matrix.

18 $$\begin{aligned}- \partial _\tau J(\tau , {\textbf{x}}) {\mathbb {I}}^{(r)}_4 &= \epsilon _r mc \gamma ^\mu u_\mu {\mathbb {I}}^{(r)}_4 + u^\mu {\mathbb {I}}^{(r)}_4 \left( \partial _\mu J(\tau , {\textbf{x}}) + q A_\mu (\tau , {\textbf{x}}) \right) {\mathbb {I}}^{(r)}_4 + \\  & \quad + \frac{1}{2} \sigma ^\mu \sigma ^\mu g_{\mu \mu } \partial ^\mu \partial _\mu J(\tau , {\textbf{x}}) {\mathbb {I}}^{(r)}_4, \quad r=1,2,3,4 \end{aligned}$$

We should note that if the first term in Eq. (14) was not linearized, then we would have four identical equations for $r=1,2,3,4$. Another observation is that we can rewrite $\partial _\mu J(\tau , {\textbf{x}}) {\mathbb {I}}^{(r)}_4=\partial _\tau (J(\tau , {\textbf{x}}) {\mathbb {I}}^{(r)}_4)=\partial _\tau \varvec{J}^{(r)}(\tau , {\textbf{x}})$ and $mc \gamma ^\mu u_\mu {\mathbb {I}}^{(r)}_4=mc \gamma ^\mu (u_\mu {\mathbb {I}}^{(r)}_4)=mc \gamma ^\mu \varvec{u}^\mu _{(r)}$. This allows us to treat both $\varvec{J}$ and the $\varvec{u}_\mu$ as four-component column vectors. Then Eq. (18) can be written as:

19 $$\begin{aligned}- \partial _\tau \varvec{J}^{(r)}(\tau , {\textbf{x}}) &= \epsilon _r mc \gamma ^\mu \varvec{u}^{(r)}_\mu + \varvec{u}^\mu \left( \partial _\mu \varvec{J}^{(r)}(\tau , {\textbf{x}}) + q A_\mu (\tau , {\textbf{x}}) \right)+ \\  & \quad + \frac{1}{2} \sigma ^\mu \sigma ^\mu g_{\mu \mu } \partial ^\mu \partial _\mu \varvec{J}^{(r)}(\tau , {\textbf{x}}), \quad r=1,2,3,4. \end{aligned}$$

As Nelson proposed in^6^, the variance of the Brownian motion of the particle is equal to the ratio of Planck constant to the mass of the particle. That is why we express the square of the diffusion coefficient in (2) to be proportional to the $\frac{\hbar }{m}$.

20 $$\begin{aligned} g_{\mu \mu } \sigma ^\mu \sigma ^\mu = - \frac{2 i \epsilon _r \hbar }{m}, \quad \mu =0..3 \end{aligned}$$

In the original idea proposed by Nelson, the imaginary unit, the factor 2, and the temporal diffusion coefficient were missing from the equation above. Later, we will demonstrate that the introduction of the imaginary unit and the factor 2 in the equation above is necessary for the linearization of Eq. (15). This serves as another key contribution of the current paper, explaining the existence of the imaginary unit in quantum mechanics equations. However, it is important to note that the imaginary diffusion coefficient will result in a complex stochastic Eq. (2), as we will show in Sect. “Diffusion coefficients of the stochastic motion”.

If we substitute Eqs. (20) and (13) into (19) we obtain:

21 $$\begin{aligned}- \partial _\tau \varvec{J}^{(r)}(\tau , {\textbf{x}}) &=  - \epsilon _r mc \gamma ^\mu \frac{1}{m} \left( \partial _\mu \varvec{J}^{(r)}(\tau , \textbf{x}) + q A_\mu \right) - \frac{i \epsilon _r \hbar }{m} \partial ^\mu \partial _\mu \varvec{J}^{(r)}(\tau , {\textbf{x}})- \\&\quad - \frac{1}{m} \left( \partial ^\mu \varvec{J}^{(r)}(\tau , {\textbf{x}}) + q A^\mu \right) \left( \partial _\mu \varvec{J}^{(r)}(\tau , {\textbf{x}}) + q A_\mu \right) , \quad r=1,2,3,4. \end{aligned}$$

Expanding the last term of the above equation we obtain:

22 $$\begin{aligned}- \partial _\tau \varvec{J}^{(r)}(\tau , {\textbf{x}}) &= - \epsilon _r mc \gamma ^\mu \frac{1}{m} \left( \partial _\mu \varvec{J}^{(r)}(\tau , \textbf{x}) + q A_\mu \right) - \frac{i \epsilon _r \hbar }{m} \partial ^\mu \partial _\mu \varvec{J^{(r)}}(\tau , {\textbf{x}}) - \\&\quad - \frac{1}{m} \partial ^\mu \varvec{J}^{(r)}(\tau , {\textbf{x}}) \partial _\mu \varvec{J}^{(r)}(\tau , {\textbf{x}}) - 2 \frac{1}{m} \partial ^\mu \varvec{J}^{(r)}(\tau , {\textbf{x}}) q A_\mu - \frac{1}{m} q^2 A^\mu A_\mu , \, r=1,2,3,4. \end{aligned}$$

Let us introduce a new function ${\tilde{J}}(\tau , {\textbf{x}})$:

23 $$\begin{aligned} \varvec{J}^{(r)}(\tau , {\textbf{x}}) = -i \epsilon _r \hbar {\tilde{J}}(\tau , {\textbf{x}}) {\mathbb {I}}^{(r)}_4, \quad r=1,2,3,4. \end{aligned}$$

If we use this new function in Eq. (13), it will make the equation asymmetric with respect to $\epsilon _r$. That is why let us denote $q=\epsilon _r e$ and rewrite Eq. (13) as:

24 $$\begin{aligned} \varvec{u}^{(r)}_\mu = - \frac{1}{m} \left( -i \epsilon _r \hbar \partial _\mu {\tilde{J}}(\tau , {\textbf{x}}) + \epsilon _r e A_\mu (\tau , {\textbf{x}}) \right) {\mathbb {I}}^{(r)}_4, r=1,2,3,4. \end{aligned}$$

This result is quite interesting because it tells us that the last two equations in the initial set of Eq. (19) with $r=3,4$ are for particles with the opposite sign of the charge compared to the first two equations with $r=1,2$.

Substituting Eq. (23) into Eq. (22), and then multipling both sides by *m*, we obtain:

25 $$\begin{aligned}i \hbar m \partial _\tau {\tilde{J}}(\tau , {\textbf{x}}) &= m c \gamma ^\mu \left( i \hbar \partial _\mu {\tilde{J}}(\tau , {\textbf{x}}) - e A_\mu \right) + \\ & \quad + \hbar ^2\left( \partial ^\mu {\tilde{J}}(\tau , {\textbf{x}}) \partial _\mu {\tilde{J}}(\tau , {\textbf{x}}) + \partial ^\mu \partial _\mu {\tilde{J}}(\tau , {\textbf{x}}) \right) + 2 i \hbar \partial ^\mu {\tilde{J}}(\tau , {\textbf{x}}) e A_\mu - e^2 A^\mu A_\mu . \end{aligned}$$

Using (Hopf-Cole) logarithmic transformation, defined by ${\tilde{J}}(\tau , \textbf{x})=log(\phi (\tau ,{\textbf{x}}))$, we can linearize the HJB equation through the following calculation:

26 $$\partial ^\mu {\tilde{J}}(\tau , {\textbf{x}}) \partial _\mu {\tilde{J}}(\tau , {\textbf{x}}) + \partial ^\mu \partial _\mu {\tilde{J}}(\tau , {\textbf{x}}) = \frac{\partial ^\mu \phi (\tau , {\textbf{x}})}{\phi (\tau , {\textbf{x}})} \frac{\partial _\mu \phi (\tau , {\textbf{x}})}{\phi (\tau , {\textbf{x}})} + \partial ^\mu \left( \frac{\partial _\mu \phi (\tau , \textbf{x})}{\phi (\tau , {\textbf{x}})} \right) = \frac{\partial ^\mu \phi (\tau , {\textbf{x}})}{\phi (\tau , {\textbf{x}})} \frac{\partial _\mu \phi (\tau , {\textbf{x}})}{\phi (\tau , {\textbf{x}})} + \frac{\partial ^\mu \partial _\mu \phi (\tau , {\textbf{x}})}{\phi (\tau , {\textbf{x}})} - \frac{\partial ^\mu \phi (\tau , {\textbf{x}}) \partial _\mu \phi (\tau , {\textbf{x}})}{\phi (\tau , {\textbf{x}})^2} = \frac{\partial ^\mu \partial _\mu \phi (\tau , \textbf{x})}{\phi (\tau , {\textbf{x}})}.$$

Now, it becomes clear that the presence of the imaginary unit in the diffusion coefficient (20) and the imaginary unit in (23) are a consequence of the need to linearize the HJB equation.

The explicit equation for $\phi (\tau ,{\textbf{x}})$ is:

27 $$\begin{aligned} \varvec{\phi }^{(r)}(\tau ,{\textbf{x}}) = \phi (\tau ,{\textbf{x}}) {\mathbb {I}}^{(r)}_4=e^{-\frac{\epsilon _r}{i \hbar } J(\tau , \textbf{x})} {\mathbb {I}}^{(r)}_4, \quad r=1,2,3,4. \end{aligned}$$

later we will discuss what is the physical meaning of this function.

The above substitution transforms the HJB into a linear equation:

28 $$\begin{aligned}i \hbar m \frac{\partial _\tau \phi (\tau , {\textbf{x}})}{\phi (\tau , {\textbf{x}})} = m c \left( i \hbar \gamma ^\mu \frac{\partial _\mu \phi (\tau , {\textbf{x}})}{\phi (\tau , {\textbf{x}})} - e \gamma ^\mu A_\mu \right) + \hbar ^2 \frac{\partial ^\mu \partial _\mu \phi (\tau , \textbf{x})}{\phi (\tau , {\textbf{x}})} + 2 e A^\mu i \hbar \frac{\partial _\mu \phi (\tau , {\textbf{x}})}{\phi (\tau , {\textbf{x}})} - e^2 A^\mu A_\mu . \end{aligned}$$

Multiplying both sides of Eq. (28) by $\phi (\tau , {\textbf{x}})$, finally we obtain:

29 $$\begin{aligned}i \hbar m \partial _\tau \phi (\tau , {\textbf{x}})& = m c \left( i \hbar \gamma ^\mu \partial _\mu \phi (\tau , {\textbf{x}}) - e \gamma ^\mu A_\mu \phi (\tau , {\textbf{x}}) \right) + \hbar ^2 \partial ^\mu \partial _\mu \phi (\tau , {\textbf{x}})+ \\ & \quad + 2 e A^\mu i \hbar \partial _\mu \phi (\tau , {\textbf{x}}) - e^2 A^\mu A_\mu \phi (\tau , {\textbf{x}}). \end{aligned}$$

We are going to prove that Eq. (29) is equivalent to:

30 $$\begin{aligned} i \hbar m \partial _\tau \phi (\tau , {\textbf{x}}) = \left( i \hbar \gamma ^\nu \partial _\nu - e \gamma ^\nu A_\nu - mc \right) \left( - i \hbar \gamma ^\mu \partial _\mu \phi (\tau , {\textbf{x}}) + e \gamma ^\mu A_\mu \phi (\tau , {\textbf{x}}) \right) . \end{aligned}$$

After expanding the brackets we obtain:

31 $$\begin{aligned}i \hbar m \partial _\tau \phi (\tau , {\textbf{x}})&= mc \left( i \hbar \gamma ^\mu \partial _\mu \phi (\tau , {\textbf{x}}) - e \gamma ^\mu A_\mu \phi (\tau , {\textbf{x}}) \right) + \hbar ^2 \gamma ^\nu \partial _\nu \gamma ^\mu \partial _\mu \phi (\tau , {\textbf{x}}) + \\&\quad + i \hbar \gamma ^\nu \partial _\nu \left( e \gamma ^\mu A_\mu \phi (\tau , {\textbf{x}}) \right) + e \gamma ^\nu A_\nu i \hbar \gamma ^\mu \partial _\mu \phi (\tau , {\textbf{x}}) - e \gamma ^\nu A_\nu e \gamma ^\mu A_\mu \phi (\tau , {\textbf{x}}). \end{aligned}$$

To simplify the above equation we will use the following relations:

32 $$\begin{aligned}\gamma ^\mu \partial _\mu \gamma ^\nu \partial _\nu = \partial ^\mu \partial _\mu \qquad \gamma ^\mu \partial _\mu \gamma ^\nu A_\nu = \partial _\mu A^\mu \qquad \gamma ^\mu A_\mu \gamma ^\nu A_\nu = A^\mu A_\mu \end{aligned}$$

To prove that $\gamma ^\mu B_\mu \gamma ^\nu B_\nu = B^\mu B_\mu$ lets sum two times $\gamma ^\mu B_\mu \gamma ^\nu B_\nu$ and take one half of this sum. Also let us use the fact that we can change the name of summation indexes, and the property $\gamma ^\mu \gamma ^\nu + \gamma ^\nu \gamma ^\mu = g^{\mu \nu }$: $\gamma ^\mu B_\mu \gamma ^\nu B_\nu = \frac{1}{2}( \gamma ^\mu B_\mu \gamma ^\nu B_\nu + \gamma ^\nu B_\nu \gamma ^\mu \partial _\mu ) = \frac{1}{2}( \gamma ^\mu \gamma ^\nu + \gamma ^\nu \gamma ^\mu ) B_\mu B_\nu = \frac{1}{2} 2 g^{\mu \nu } B_\mu B_\mu = B^\mu B_\mu$.

33 $$\begin{aligned}i \hbar m \partial _\tau \phi (\tau , {\textbf{x}})&= mc \left( i \hbar \gamma ^\mu \partial _\mu \phi (\tau , {\textbf{x}}) - e \gamma ^\mu A_\mu \phi (\tau , {\textbf{x}}) \right) + \hbar ^2 \partial ^\mu \partial _\mu \phi (\tau , {\textbf{x}}) +\\&\quad + i \hbar e ( \partial _\mu A^\mu ) \phi (\tau , {\textbf{x}}) + i \hbar e A^\mu \partial _\mu \phi (\tau , {\textbf{x}}) + e A^\mu i \hbar \partial _\mu \phi (\tau , {\textbf{x}}) - e^2 A^\mu A_\mu \phi (\tau , {\textbf{x}}). \end{aligned}$$

If we use the Lorenz gauge condition^32^ $\partial _\mu A^\mu =0$, the Eq. (33) will turn into Eq. (29), which proves that both Eqs. (29) and (31) are equivalent.

Let us introduce the four-component column vector function:

34 $$\begin{aligned} \varvec{\psi }(\tau , {\textbf{x}}) = - \frac{i \hbar }{mc} \gamma ^\mu \partial _\mu \varvec{\phi }(\tau , {\textbf{x}}) + \frac{e}{m c} \gamma ^\mu A_\mu \varvec{\phi }(\tau , {\textbf{x}}), \end{aligned}$$

the factor $\frac{1}{m c}$ is introduced to ensure that $\varvec{\psi }(\tau , {\textbf{x}})$ has dimensionless units.

If we divide the Eq. (30) by *mc*, then we obtain the following equation for any *r*:

35 $$\begin{aligned} \frac{i \hbar }{c} \partial _\tau \varvec{\phi }(\tau , {\textbf{x}}) = \left( i \hbar \gamma ^\nu \partial _\nu - e \gamma ^\nu A_\nu - mc \right) \varvec{\psi }(\tau , {\textbf{x}}). \end{aligned}$$

It should be noted that the Dirac equation is stationary equation when one set the partial derivative with respect to proper time to zero.

36 $$\begin{aligned}\left( i \hbar \gamma ^\nu \partial _\nu - e \gamma ^\nu A_\nu - mc \right) \varvec{\psi }({\textbf{x}}) = 0 \end{aligned}$$

Where $\varvec{\psi }({\textbf{x}})$ is the stationary solution that does not depend on the proper time. As it can be seen from (36) that $\varvec{\psi }({\textbf{x}})$ is the Dirac spinor, which from (34) becomes:

37 $$\begin{aligned} \varvec{\psi }({\textbf{x}}) = - \frac{1}{m c} i \hbar \gamma ^\mu \partial _\mu \varvec{\phi }({\textbf{x}}) + \frac{1}{m c} e \gamma ^\mu A_\mu \varvec{\phi }({\textbf{x}}). \end{aligned}$$

Note that derivation of Dirac equation presented in this paper does not derive the equation with the negative mass $-m$. Even some recent theoretical attempt to recover the idea of negative mass^33^, until now there is no experimental evidence of particle with negative mass.

## Dirac spinor and the wave function

In the previous section, we expressed the Dirac spinor in Eq. (37) as a function of $\phi ({\textbf{x}})$, which is a stationary solution of the HJB equation, and its derivative $\partial _\mu \phi ({\textbf{x}})$.

As any other solution $\phi (\tau , {\textbf{x}})$ of HJB equation, the stationary solution $\phi ({\textbf{x}})$ should also satisfy the Eq. (27):

38 $$\begin{aligned} \varvec{\phi }^{(r)}({\textbf{x}})=\phi ({\textbf{x}}) {\mathbb {I}}^{(r)}_4=e^{-\frac{\epsilon _r}{i \hbar } J({\textbf{x}})} {\mathbb {I}}^{(r)}_4, r=1,2,3,4. \end{aligned}$$

The function $\phi ({\textbf{x}})$ is identical as the wave function in the path integral formulation of quantum mechanics proposed by Feynman^34^. That is why $|\phi ({\textbf{x}})|^2$ is the probability density of the system to be in spacetime position ${\textbf{x}}$. This conclusion aligns with the work of Papiez^16^. Note that here we do not give probabilistic physical interpretation of function $\phi (\tau , {\textbf{x}})$, because this function is object of the stochastic optimal control theory, and it is not an object of path integral formulation of quantum mechanics. The connection of both theories is via the stationary solution of HJB.

## Proper time in Dirac equation

In his paper, Fock employed a representation of the Dirac spinor (see Chapter 37-2, Section II, in^35^) that appears strikingly similar to the one we have derived in Eq. (37), as per our application in stochastic optimal control.

Fock was looking for a solution for the Dirac equation represented in the form $\psi =(i \hbar \gamma ^\nu \partial _\nu - e \gamma ^\nu A_\nu - m c) \Psi$, where $\Psi$ is required to satisfy a second-order equation $\hbar ^2 \Lambda \Psi = 0$. Here $\Lambda$ is an operator defined as $\Lambda = \frac{1}{\hbar ^2}(i \hbar \gamma ^\nu \partial _\nu - e \gamma ^\nu A_\nu - m c) (i \hbar \gamma ^\mu \partial _\mu - e \gamma ^\mu A_\mu - m c)$. A solution of the equation for $\Psi$ can be looked for in the form of a definite integral, $\Psi =\int _C {F d \tau }$, over the auxiliary variable $\tau$ taken between certain fixed limits (or along a specific contour in the complex $\tau$-plane). It is obvious that this equation would be satisfied if one imposes on *F* the equation, called by Fock the proper time Dirac equation, $\frac{\hbar ^2}{2 m} \Lambda F = i \hbar \frac{\partial F}{\partial \tau }$ with the choice of contour *C* such that $\int _C {\frac{\partial F}{\partial \tau }} d \tau = F |_{\partial C} = 0$.

In this paper, we introduce a representation of the Dirac spinor as $\psi =-\frac{1}{mc}(i \hbar \gamma ^\nu \partial _\nu - e \gamma ^\nu A_\nu ) \phi ({\textbf{x}})$, detailed further in Eq. (37). The corresponding second-order equation that $\phi ({\textbf{x}})$ must satisfy is presented as $\hbar ^2 \Lambda \phi ({\textbf{x}}) = 0$. Here, the operator $\Lambda$ is defined by $\Lambda =\frac{1}{\hbar ^2}\left( i \hbar \gamma ^\nu \partial _\nu - e \gamma ^\nu A_\nu - mc \right) \left( - i \hbar \gamma ^\mu \partial _\mu + e \gamma ^\mu A_\mu \right)$, as elaborated in Eq. (30). Similar to Fock’s approach, we consider representing $\phi (x)$ in the form $\int _C {\phi (\tau , {\textbf{x}}) d \tau }$, with the condition that $\int _C {\frac{\partial \phi (\tau , {\textbf{x}})}{\partial \tau }} d \tau = \phi (\tau , \textbf{x}) |_{\partial C} = 0$. However, within the framework of stochastic optimal control, the parameter $\tau$ is an invariant parameter used to trace the progress of the system point in configuration space. Consequently, such a contour *C* for integration is not feasible. The feasible approach is to regard $\phi ({\textbf{x}})$ as a stationary solution of the time-dependent second-order differential equation $\frac{\hbar ^2}{2 m} \Lambda \phi (\tau , {\textbf{x}}) = i \hbar \partial _\tau \phi (\tau , {\textbf{x}})$, as outlined in Eq. (29).

Even though we cannot directly apply Fock’s technique to the stochastic optimal control case, we can still identify Eq. (33) or the equivalent Eq. (29) as the proper time Dirac equation. This is because, by definition, the variable $\tau$ in these equations represents the proper time.

### Physical interpretation of the stationary solution of HJB equation

To accurately interpret the stationary solution of the Hamilton–Jacobi–Bellman (HJB) equation and the associated proper time Dirac equation as derived in Eq. (29), a detailed analysis of this equation is essential. One important observation is that we need to solve the HJB equation reverse in time. This means that, given the boundary condition at the future time moment $\tau _f$ and spacetime coordinate $x_{\tau _f}$, we aim to find the solution of this equation for an earlier time moment $\tau$ and coordinate $x_\tau$. This time-reversal approach is typical for the dynamic programming^26^, as incorporated in the derivation of the HJB equation.

It is required that $x_\tau$ be a four-vector of real numbers. However, there is no requirement for the four-coordinates corresponding to times greater than $\tau$ to be real numbers, see the discussion in^16^; they can be a four-vector of complex numbers. This implies that no matter what the complex four-coordinates and four-velocities of the particle are at the time moments greater than $\tau$ when it moves backward in time, it should arrive at the initial time $\tau$ with the real four-coordinate $x_\tau$.

As far as $\tau _f$ being any arbitrary final moment in time, we can choose $\tau _f$ to be sufficiently large so that when the particles propagate backward in time, their motion becomes stationary and ceases to depend on time.

In all physical theories, the aim is to identify an equation that describes how a system evolves over time. Essentially, this means determining the state of the system at successive time moments based on its state at previous moments. The stochastic optimal control theory of quantum mechanics also allows for this traditional approach, as demonstrated in this paper through the derivation of the Dirac equation. However, the theory also offers an alternative approach. To determine the wave function for every spacetime coordinate $x_\tau$, we must choose some future proper time $\tau _f$. We then backtrack all possible complex stochastic trajectories of the particle to the present time $\tau$ and real four-coordinates $x_\tau$, aiming to find the optimal trajectories that minimize the expected value of the action. It appears that the current state of the system is determined by all possible optimal future trajectories of the particle.

## Plane wave solutions of the Dirac equation

Let us consider a particle with mass *m* that moves with velocity $u_\mu$ in free space, where $A_\mu =0$.

If we multiply both sides of Eq. (13) by *m*, we find that the particle momentum is given by $p_\mu = - \partial _\mu J(x)$. A solution to this equation is $J(x) = - p \cdot x$. Substituting *J*(*x*) into Eq. (38), we obtain the equation for a plane wave, which describes the particle: $\varvec{\phi }^{(r)}(x) = e^{-\frac{i \epsilon _r}{\hbar } p \cdot x} {\mathbb {I}}^{(r)}_4, r=1,2,3,4$. We can calculate the derivative $\partial _\mu \varvec{\phi }^{(r)}(x) = \frac{\epsilon _r}{i \hbar } p_\mu e^{- \frac{i \epsilon _r}{\hbar } (p \cdot x)} {\mathbb {I}}^{(r)}_4, r=1,2,3,4$ and then substitute this result into Eq. (37).

39 $$\begin{aligned} &\varvec{\psi }^{(r)}(x) = - \frac{\epsilon _r}{m c} \gamma ^\mu p_\mu e^{- \frac{i\epsilon _r}{\hbar }( p \cdot x)} {\mathbb {I}}^{(r)}_4, \quad r=1,2,3,4 \end{aligned}$$

Now we will prove that $\varvec{\psi }^{(r)}(x), r=1,2,3,4$, satisfies the Dirac Eq. (36).

40 $$\begin{aligned}- (i \hbar \gamma ^\mu \partial _\mu - m c) \frac{\epsilon _r}{m c} \gamma ^\mu p_\mu e^{- \frac{i \epsilon _r}{\hbar }( p \cdot x)} {\mathbb {I}}^{(r)}_4 = (\gamma ^\mu p_\mu - \epsilon _r m c) e^{- \frac{i \epsilon _r}{\hbar }( p \cdot x)} \epsilon _r {\mathbb {I}}^{(r)}_4 = 0 \end{aligned}$$

The last equation equals zero due to Eqs. (9) and (14). This demonstrates that $\varvec{\psi }^{(r)}(x)$ from Eq. (39) are indeed solutions of the Dirac Eq. (36).

## Diffusion coefficients of the stochastic motion

In Sect. “Derivation of Dirac equation”, we derived the Dirac equation by linearizing the stochastic HJB equations, necessitating the use of complex diffusion coefficients (refer to Eq. (20)). The works of Papiez^16,20^ and Lindgren et al.^12^ also employ optimal control theory in conjunction with complex diffusion coefficients. In contrast, earlier variational approaches to deriving stochastic mechanics^17,36,37^ do not rely on complex numbers but utilize two control parameters: the forward ($b_{+}=v_\rho + u_\rho$) and backward ($b_{-}=v_\rho - u_\rho$) velocities in the variational procedures.

In their work on Complex Mechanics^38^, Yang et al. prove that the current velocity $v_\rho$ and the osmotic velocity $u_\rho$, as defined in stochastic mechanics of Nelson, are equivalent to the real part and negative imaginary part, respectively, of the complex velocity evaluated on the real axis. Thus, one can consider the complex diffusion coefficient and related complex stochastic equations of motion as a theoretical construct to simplify the derivation of quantum mechanics equations from stochastic optimal control theory, or it can be viewed as an intrinsic aspect of reality, subject to interpretation.

It should be noted that, in contrast to non-relativistic Complex Mechanics as discussed in Yang et al.^38^ and other works utilizing a complex diffusion coefficient $\nu =-i \hbar / m$^3,7,12,16^, in the relativistic case the diffusion coefficient is two times larger, $\nu =-2 i \epsilon _r \hbar / m$ (see Eq. (20)). This increase is a consequence of the linearization performed on the kinetic term of the Lagrangian (see Eq. (14)). Note also that the sign of complex diffusion coefficient is opposite for the equations $r=1,2$ and $r=3,4$ that describes particles with opposite changes as per Eq. (24).

If we take the square root of both sides of Eq. (20), we obtain the following equation for the diffusion coefficient:

41 $$\begin{aligned} \sigma ^\mu =\pm \sqrt{\frac{2 g_{\mu \mu } \epsilon _r i \hbar }{m}},\quad \mu =0..3. \end{aligned}$$

If we use the identity $\sqrt{\pm i}=\frac{1}{\sqrt{2}}(1\pm i)$, we finally obtain:

42 $$\begin{aligned} \sigma ^\mu =\pm \ (1 + \epsilon _r g_{\mu \mu } i) \sqrt{\frac{\hbar }{m}},\quad \mu =0..3. \end{aligned}$$

If we rewrite the equation of stochastic motion (2) using Eq. (42) we obtain:

43 $$\begin{aligned} d x_\mu =u_\mu ds + (1 + \epsilon _r g_{\mu \mu } i){\sqrt{\frac{\hbar }{m}}} dW_\mu , \quad \mu =0..3. \end{aligned}$$

The presence of complex diffusion coefficients and complex four-velocity in the stochastic equation of motion (2) necessitates that the particle’s four-coordinates also be complex-valued. Taking the real and imaginary parts of the four-coordinates allows us to formulate the equations of stochastic motion (2) for both real and imaginary spacetime coordinates.

44 $$\begin{aligned} {\text {Re}}(d x_\mu )= & {} {\text {Re}}(u_\mu ) ds + \sqrt{\frac{\hbar }{m}} dW_\mu , \quad \mu =0..3 \end{aligned}$$

45 $$\begin{aligned} {\text {Im}}(d x_\mu )= & {} {\text {Im}}(u_\mu ) ds + \epsilon _r g_{\mu \mu } \sqrt{\frac{\hbar }{m}} dW_\mu , \quad \mu =0..3 \end{aligned}$$

The coordinates of the test particles, both the real and imaginary parts, can be treated separately as coordinates in Minkowski spacetime in the same way as it is done in^38^.

## Conclusion

The present work derives the Dirac equation from the stochastic optimal control theory and provides representation of Dirac spinor using the solution of Hamilton–Jacobi–Bellman (HJB) equation.

We proposed a linearization of the Lagrangian in the HJB equation, which yields four independent equations. The last two of which are for particles with opposite charges.

It has been demonstrated that in the case of relativistic theory, the complex diffusion coefficient is twice as large as in the non-relativistic theory, with an opposite sign for the oppositely charged particle.

We also proposed a physical interpretation of the proper time Dirac equation in terms of stochastic optimal control theory that leads to the observation that the current state of the system is determined by all possible optimal future trajectories of the particle.

Even though the stochastic mechanics has some difficulties^39^ in explaining the locality (see Chapter 23 in^15^) and entanglement (see Chapter 10 in^40^) the present paper makes one step further of explaining other feature of the theory that was not revealed until now.

### Supplementary Information

Supplementary Information.

## Data Availability

All data generated or analysed during this study are included in this published article and its supplementary information files.
