# Supplementary material for: Derivation of Dirac equation from the stochastic optimal control principles of quantum mechanics
Source: Sci Rep. 2024 Mar 18;14:6507. doi: 10.1038/s41598-024-56582-5 (PMC10948848; doi:10.1038/s41598-024-56582-5)
Supplement: Supplementary file 1 — Supplementary Information. [file 41598_2024_56582_MOESM1_ESM.pdf]

Derivation of the stochastic Hamilton-Jacobi-Bellman equation

Vasil Yordanov
Sofia University St. Kliment Ohridski
v.yordanov@phys.uni-sofia.bg

December 2, 2023

Abstract

In the present paper, we provide a detailed derivation of the stochastic Hamilton–Jacobi–Bellman (HJB) equation.

1 Introduction

In this paper, we will derive the stochastic Hamilton–Jacobi–Bellman (HJB) equation. The derivation presented here is inspired by the following papers: [1, 2, 3].

2 Derivation of the stochastic HJB equation

The stochastic equation of motion of the particle is:

$$dx_\mu = u_\mu ds + \sigma_\mu dW_\mu, \quad \mu = 0..3, \tag{2.1}$$

where x_μ are the spacetime coordinates $\mathbf{x}$ of the particle, and u_μ are the components of the four-velocity $\mathbf{u}$.

The action is postulated as minimum of the expected value of stochastic action:

$$S(\mathbf{x}_i, \mathbf{u}(\tau_i \to \tau_f)) = \min_{\mathbf{u}(\tau_i \to \tau_f)} \left\langle \int_{\tau_i}^{\tau_f} ds\, \mathcal{L}(\mathbf{x}(s), \mathbf{u}(s), s) \right\rangle_{\mathbf{x}_i}, \tag{2.2}$$

where $\mathcal{L}(\mathbf{x}(s), \mathbf{u}(s), s)$ is the Lagrangian of the test particle, which is a function of the control policy $\mathbf{u}(s)$ and four-coordinates $\mathbf{x}(s)$ at proper time s . The subscript $\mathbf{x}_i$ on the expectation value means that the expectation is over all stochastic trajectories that start at $\mathbf{x}_i$.

The task of optimal control theory [4] is to find the control $\mathbf{u}(s)$, $\tau_i < s < \tau_f$, denoted as $\mathbf{u}(\tau_i \to \tau_f)$, that minimizes the expected value of the action $S(\mathbf{x}_i, \mathbf{u}(\tau_i \to \tau_f))$.

We introduce the optimal cost-to-go function for any intermediate proper time τ, where $\tau_i < \tau < \tau_f$:

$$J(\tau, \mathbf{x}_\tau) = \min_{\mathbf{u}(\tau \to \tau_f)} \left\langle \int_{\tau}^{\tau_f} ds\, \mathcal{L}(s, \mathbf{x}_s, \mathbf{u}_s) \right\rangle_{\mathbf{x}_\tau} \tag{2.3}$$

By definition, the action $S(\mathbf{x}_i, \mathbf{u}(\tau_i \to \tau_f))$ is equal to the cost-to-go function $J(\tau_i, \mathbf{x}_{\tau_i})$ at the initial proper time and spacetime coordinate:

$$S(\mathbf{x}_i, \mathbf{u}(\tau_i \to \tau_f)) = J(\tau_i, \mathbf{x}_{\tau_i}) \tag{2.4}$$

We can rewrite recursive formula for $J(\tau, \mathbf{x}_\tau)$ for any intermediate time τ', where $\tau < \tau' < \tau_f$:

$$\begin{aligned} J(\tau, \mathbf{x}_\tau) &= \min_{\mathbf{u}(\tau\to\tau')} \left\langle \int_\tau^{\tau'} ds\, \mathcal{L}(s, \mathbf{x}_s, \mathbf{u}_s) + \int_{\tau'}^{\tau_f} ds\, \mathcal{L}(s, \mathbf{x}_s, \mathbf{u}_s) \right\rangle_{\mathbf{x}_\tau} \\ &= \min_{\mathbf{u}(\tau\to\tau')} \left\langle \int_\tau^{\tau'} ds\, \mathcal{L}(s, \mathbf{x}_s, \mathbf{u}_s) + \min_{\mathbf{u}(\tau'\to\tau_f)} \left\langle \int_{\tau'}^{\tau_f} ds\, \mathcal{L}(s, \mathbf{x}_s, \mathbf{u}_s) \right\rangle_{\mathbf{x}_{\tau'}} \right\rangle_{\mathbf{x}_\tau} \\ &= \min_{\mathbf{u}(\tau\to\tau')} \left\langle \int_\tau^{\tau'} ds\, \mathcal{L}(s, \mathbf{x}_s, \mathbf{u}_s) + J(\tau', \mathbf{x}_{\tau'}) \right\rangle_{\mathbf{x}_\tau} . \end{aligned} \tag{2.5}$$

In above equation we split the minimization over two intervals. These are not independent, because the second minimization is conditioned on the starting value $x_{\tau'}$, which depends on the outcome of the first minimization.

If τ' is a small increment of τ, $\tau' = \tau + d\tau$ then:

$$J(\tau, x_\tau) = \min_{\mathbf{u}(\tau\to\tau+d\tau)} \langle \mathcal{L}(\tau, \mathbf{x}_\tau, \mathbf{u}_\tau) d\tau + J(\tau + d\tau, \mathbf{x}_{\tau+d\tau}) \rangle_{\mathbf{x}_\tau} \tag{2.6}$$

We must take a Taylor expansion of J in $d\tau$ and $d\mathbf{x}$. However, since $\langle d\mathbf{x}^2 \rangle = \sigma^2 d\tau$ is of order $d\tau$, we must expand up to order $d\mathbf{x}^2$:

$$\begin{aligned} &\langle J(\tau + d\tau, \mathbf{x}_{\tau+d\tau}) \rangle_{\mathbf{x}_\tau} = \int d\mathbf{x}_{\tau+d\tau} \mathcal{N}(\mathbf{x}_{\tau+d\tau} | \mathbf{x}_\tau, \sigma d\tau) J(\tau + d\tau, \mathbf{x}_{\tau+d\tau}) \\ &= \int d\mathbf{x}_{t+d\tau} \mathcal{N}(\mathbf{x}_{t+d\tau} | \mathbf{x}_\tau, \sigma d\tau)(J(\tau, \mathbf{x}) + d\tau \partial_\tau J(\tau, \mathbf{x}_\tau) + dx^\mu \partial_\mu J(\tau, \mathbf{x}_\tau) + dx^\mu dx^\nu \frac{1}{2} \partial_{\mu\nu} J(\tau, \mathbf{x}_\tau)) \\ &= J(\tau, \mathbf{x}) + d\tau \partial_\tau J(\tau, \mathbf{x}_\tau) + \int dx_{t+d\tau} \mathcal{N}(\mathbf{x}_{t+d\tau} | \mathbf{x}_\tau, \sigma d\tau)(dx^\mu \partial_\mu J(\tau, \mathbf{x}_\tau) + dx^\mu dx^\nu \frac{1}{2} \partial_{\mu\nu} J(\tau, \mathbf{x}_\tau)) \\ &= J(\tau, \mathbf{x}) + d\tau \partial_\tau J(\tau, \mathbf{x}_\tau) + \langle dx^\mu \rangle \partial_\mu J(\tau, \mathbf{x}_\tau) + \frac{1}{2} \langle dx^\nu dx^\mu \rangle \partial_{\nu\mu} J(\tau, \mathbf{x}_\tau) \end{aligned} \tag{2.7}$$

Here $\mathcal{N}(\mathbf{x}_{\tau+d\tau} | \mathbf{x}_\tau, \sigma d\tau)$ is the conditional probability starting from state $\mathbf{x}_\tau$ to end up in state $\mathbf{x}_{\tau+d\tau}$. The integration is over the entire spacetime. In the above equation we also use the notation $\partial_\mu J(\tau, \mathbf{x})$ as partial derivative with respect to x_μ.

We can calculate $\langle dx^\mu \rangle$ using equations (2.1):

$$\begin{aligned} &\langle dx^\mu \rangle = \int d\mathbf{x}_{\tau+d\tau} \mathcal{N}(\mathbf{x}_{\tau+d\tau} | \mathbf{x}_\tau, \sigma^\mu d\tau) dx^\mu \\ &= \int d\mathbf{x}_{\tau+d\tau} \mathcal{N}(\mathbf{x}_{\tau+d\tau} | \mathbf{x}_\tau, \sigma d\tau)(\mathbf{u}^\mu d\tau + \sigma^\mu dW^\mu) \\ &= \mathbf{u}^\mu d\tau \int d\mathbf{x}_{t+d\tau} \mathcal{N}(\mathbf{x}_{\tau+d\tau} | \mathbf{x}_\tau, \sigma^\mu d\tau) + \int d\mathbf{x}_{\tau+d\tau} \mathcal{N}(\mathbf{x}_{\tau+d\tau} | \mathbf{x}_\tau, \sigma^\mu d\tau) \sigma^\mu dW^\mu \end{aligned} \tag{2.8}$$

From where:

$$\langle dx^\mu \rangle = u^\mu d\tau \tag{2.9}$$

In similar way we calculate $\langle dx^\nu dx^\mu \rangle$:

$$\begin{aligned}
\langle dx^\nu dx^\mu \rangle &= \int d\mathbf{x}_{\tau+d\tau} \mathcal{N}(\mathbf{x}_{\tau+d\tau}|\mathbf{x}_\tau, \sigma^\mu d\tau) dx^\mu dx^\nu \\
&= \int d\mathbf{x}_{\tau+d\tau} \mathcal{N}(\mathbf{x}_{\tau+d\tau}|\mathbf{x}_\tau, \sigma^\mu d\tau)(u^\mu d\tau + \sigma^\mu dW^\mu)(u^\nu d\tau + \sigma^\nu dW^\nu) \\
&= \int d\mathbf{x}_{\tau+d\tau} \mathcal{N}(\mathbf{x}_{\tau+d\tau}|\mathbf{x}_\tau, \sigma^\mu d\tau)(u^\mu u^\nu d^2\tau + u^\mu \sigma^\nu d\tau dW^\nu + \sigma^\mu dW^\mu u^\nu d\tau + \sigma^\mu dW^\mu \sigma^\nu dW^\nu)
\end{aligned} \tag{2.10}$$

From where we derive:

$$\langle dx^\nu dx^\mu \rangle = 0, \mu \neq \nu, \quad \left\langle (dx^\mu)^2 \right\rangle = \sigma^\mu \sigma^\mu d\tau \tag{2.11}$$

After substituting the above equations in (2.7) we derive the stochastic HJB equation:

$$-\partial_\tau J(\tau, \mathbf{x}) = \min_{\mathbf{u}} \left(\mathcal{L}(\tau, \mathbf{x}, \mathbf{u}) + u^\mu \partial_\mu J(\tau, \mathbf{x}) + \frac{1}{2} \sum_{\mu=0}^{3} \sigma^\mu \sigma^\mu \partial_{\mu\mu} J(\tau, \mathbf{x}_\tau) \right) \tag{2.12}$$

It is clear the from its definition of $J(\tau, x_\tau)$ that we have the following boundary condition:

$$J(\tau_f, x_{\tau_f}) = 0 \tag{2.13}$$

The optimal control at the current $\mathbf{x}$, τ, is given by:

$$u(\mathbf{x}, \tau) = \arg\min_{\mathbf{u}} \left(\mathcal{L}(\tau, \mathbf{x}, \mathbf{u}) + u^\mu \partial_\mu J(\tau, \mathbf{x}) \right). \tag{2.14}$$

Bibliography

[1] Hilbert J. Kappen. Path integrals and symmetry breaking for optimal control theory. *Journal of Statistical Mechanics: Theory and Experiment*, 2005(11):P11011–P11011, nov 2005.

[2] Hilbert Kappen. Optimal control theory and the linear bellman equation. *Neural Networks*, 08 2011.

[3] Wendell H. Fleming and H.M. Soner. *Controlled Markov Processes and Viscosity Solutions*. Stochastic Modelling and Applied Probability. Springer, New York, NY, 2 edition, 2006. Number of Pages: XVII, 429.

[4] Richard Bellman. The theory of dynamic programming. *Operations Research*, 2(3):275–285, 1954.
